# Mobile User Connectivity in Relay-Assisted Visible Light Communications

**DOI:** 10.3390/s18041125

**Published:** 2018-04-07

**Authors:** Petr Pešek, Stanislav Zvanovec, Petr Chvojka, Manav R. Bhatnagar, Zabih Ghassemlooy, Prakriti Saxena

**Affiliations:** 1 Department of Electromagnetic Field, Faculty of Electrical Engineering, Czech Technical University in Prague, 2 Technicka, 16627 Prague, Czech Republic; xzvanove@fel.cvut.cz (S.Z.); chvojpe8@fel.cvut.cz (P.C.); 2 Department of Electrical Engineering, Indian Institute of Technology Delhi, Hauz Khas, IN-110016 New Delhi, India; manav@ee.iitd.ac.in (M.R.B.); prakriti1192@gmail.com (P.S.); 3 Optical Communications Research Group, NCRLab, Faculty of Engineering and Environment, Northumbria University, NE1 8ST Newcastle upon Tyne, UK; z.ghassemlooy@northumbria.ac.uk

**Keywords:** amplify-and-forward relaying, cooperative communication, decode-and-forward relaying, visible light communications

## Abstract

In this paper, we investigate relay-assisted visible light communications (VLC) where a mobile user acts as a relay and forwards data from a transmitter to the end mobile user. We analyse the utilization of the amplify-and-forward (AF) and decode-and-forward (DF) relaying schemes. The focus of the paper is on analysis of the behavior of the mobile user acting as a relay while considering a realistic locations of the receivers and transmitters on a standard mobile phone, more specifically with two photodetectors on both sides of a mobile phone and a transmitting LED array located upright. We also investigate dependency of the bit error rate (BER) performance on the azimuth and elevation angles of the mobile relay device within a typical office environment. We provide a new analytical description of BER for AF and DF-based relays in VLC. In addition we compare AF and DF-based systems and show that DF offers a marginal improvement in the coverage area with a BER < 10^–3^ and a data rate of 100 Mb/s. Numerical results also illustrate that relay-based systems offer a significant improvement in terms of the coverage compared to direct non-line of sight VLC links.

## 1. Introduction

With the enormous growth of data traffic over wireless infrastructures due to increased demands for video and audio streaming, file sharing, data and voice over Internet protocol (VoIP) [1], the lack of available radio-frequency (RF) spectrum is becoming the limiting factor for high-speed data transmission. One possible solution to address this problem, mostly in an indoor environment at the moment, is the visible light communications (VLC) offering attractive capabilities such as vast unregulated spectrum (∼380–780 nm), inherent security and high energy efficiency [2,3].

The rapid growth of VLC is due to the development in solid-state lighting (SSL) and highly efficient white light-emitting diodes (LEDs) [4]. White LEDs offer a longer life span and much higher power efficiency (∼60–80 %) than the conventional fluorescent and incandescent lamps, as well as the possibility to be used in safe and secure applications (e.g., in hospitals, gas stations and airplanes) where RF-based technologies cannot be used [5].

Indoor VLC can be categorized into the line-of-sight (LOS) and diffuse systems. Data rates in the order of Gb/s over a very short transmission span can be achieved using LOS VLC links [6]. For instance, in [7] a 4.5 Gb/s VLC system employing carrier-less amplitude and phase (CAP) modulation and a recursive least square (RLS) based adaptive equalizer over a link span of 1.5 m was experimentally demonstrated. In [8] a data speed of 1.6 Gb/s over a 1 m link employing a combination of 16-quadrature amplitude modulation (QAM) and orthogonal frequency division multiplexing (OFDM) was reported. On the other hand, diffuse VLC systems are robust to blocking and shadowing. However, they suffer from higher losses and offer much lower data rates than LOS links due to multipath induced dispersion [9,10].

In indoor environments, the LOS path cannot always be guaranteed due to objects, people’s movement and the layout of the room [11]. To address this problem and offer seamless communications as well as to maintain an uninterrupted data access even in temporarily shadowed regions a number of solutions have been proposed [12,13]. One of the most promising techniques is the relay-assisted VLC system. Note that, the current IEEE 802.15.7 standard does not cover the relay based VLC systems, but the standard supports device discovery mechanisms in homogeneous networks [14]. In order to improve the connectivity, a full-duplex relay based VLC employing an LED lighting triangular system topology was analytically investigated in [15]. In the case of the light from an LED source mounted on the ceiling not reaching the user directly, the information can be retransmitted via a relay node (RN). In [16], the connectivity performance of mobile users based on the optical mobile relays in cooperative multi-hop VLC was investigated. An improvement in the network performance was reported by using the multi-hop scenario, which was dependent on the users’ density, coverage range ratio between hop regions, relay probabilities, and velocity of the mobile users. A number of existing works also analyzed the multi-hop VLC systems using a combination of RF and VLC links [17,18]. In order to improve the quality of service, in [17] hybrid VLC and power line communications (PLC) with a backup parallel RF link were proposed. In [18] the authors investigated the scenario, where data is transmitted from the base station to the relay via the RF link and the signal is then amplified and re-transmitted to the user over the VLC channel.

OFDM VLC over frequency-selective indoor channels was analyzed in [19] providing the first analytical statistics for pure VLC relaying using amplify-and-forward (AF) or decode-and-forward (DF) relaying schemes. In [20] a relay based DC-biased optical OFDM (DCO-OFDM) VLC was investigated for two test cases using a desk lamp and a ceiling light lamp to provide optimal power allocation and improved bit error rate (BER) performance when employing relays compared to the direct transmission.

However, none of the existing works reporting on the relay-assisted VLC systems have investigated the use of a mobile phone (MP) as a relay. In this paper, for the first time, to the best of authors’ knowledge, we provide results for performance evaluation of a relay-based VLC system employing MP as an RN for miscellaneous configurations. We give distinctive statistics of AF and DF-based relays for ceiling mounted light sources via MP, taking into account MP node orientation and a range of channel parameters. It is very important in such cases to estimate the area where such a node can be searched for, which is fully dependent on the elevation and the azimuth of MP and the required BER or the allocated optical power level. All these aspects are studied in following sections.

The rest of the paper is organized as follows. Section 2 discusses the indoor VLC channel model and the specific functionality of the MP for utilization in a relay-assisted system. Section 3 outlines a channel model for the VLC relay system and describes the cooperation techniques for the relay-assisted systems and provides analytical model for BER of AF and DF VLC. In Section 4 numerical results for the BER performance of the relay-assisted network with AF and DF modes are summarized. Finally, the summary and conclusions are given in Section 5.

## 2. Relay-VLC Deployment in the Indoor Environment

**Figure 1:**
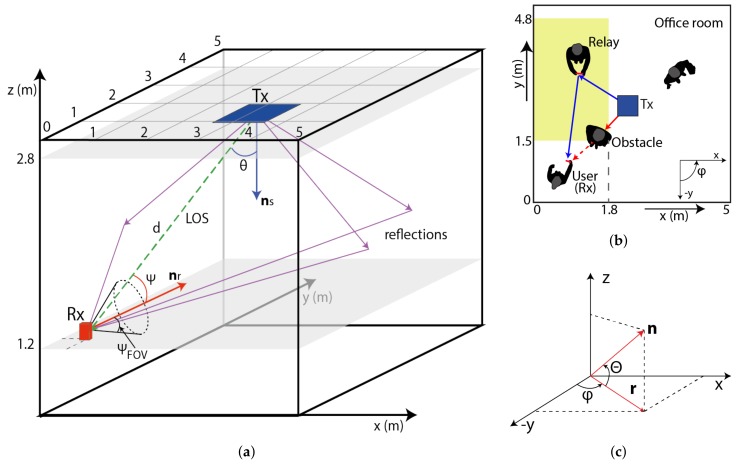
Room model: (**a**) Tx and Rx geometry model; (**b**) users’ situation in the room; and (**c**) the coordinate system.

In this paper, we consider a typical office room with a dimensions of 5 m × 5 m × 3 m with no furniture as depicted in Figure 1a. The system consists of a transmitter (Tx), which provides both illumination and data transmission, located at the center of the ceiling at the height of 2.8 m pointing downwards with an elevation angle of –90${}^{\circ }$, and a MP is used as either a receiver (Rx) or an RN. The Tx is realized an LED array with Lambertian radiation pattern. The power of LEDs is adjusted to meet the light illumination requirement of 200 to 1500 lx for an office environment as defined by International Organization for Standardization (ISO) [21].

Furthermore, we assume that a relay-based user holding a MP at the height of 1.2 m above the floor level is randomly moving around within the room. The walls, floor, and ceiling of the room are modeled as general Lambertian reflectors as in vast majority of publications [22,23]. We investigate an office environment including people where we consider shadowing between the Tx and the Rx, see Figure 1b. The LOS path between the Tx and the Rx will be blocked due to shadowing, and therefore the Tx will select a non-shadowed mobile user as an RN, which is located in the yellow area (see Figure 1b) to re-establish the link between the Tx and the Rx via the relay user. Note that, in a real environment the RN must be close to the the user, and such RN scheme would have very limiting application for considerably longer VLC connections. Here we consider an arbitrary orientation of the mobile-based RN.

The coordinates of the proposed system are depicted in Figure 1c. The unit vector ***n*** is specified in terms of conventions followed by room coordinates. The Tx and the Rx directions (i.e., elevation and azimuth angles) can be converted to unit vectors ***n_s_*** and ***n_r_***, respectively (see Figure 1a). An elevation of the Tx is an angle that ***n_s_*** makes with the xy plane, therefore if the Tx is directly pointing downwards, the elevation angle will be –90${}^{\circ }$. An azimuth angle of the Tx is defined with 0${}^{\circ }$ oriented along the negative *y*-axis in the projection of ***n_s_*** on the xy and it increases with the counter-clockwise orientation (i.e., the positive *x*-axis has an azimuth of +90${}^{\circ }$). All the key system parameters are summarized in Table 1 [21,24]. According to [25], the majority of mobile data usage (close to 80%) is in indoor environments, which are rather static, unlike the outdoor environments. Even though the location of RNs or users may change before it is initiated to retransmit the data, without loss of generality we can consider the device is stationary during the relaying process due to the slow movement of the users.

## 3. System Model

### 3.1. VLC Channel

We consider the Tx to be a monochromatic point source with a Lambertian radiation pattern. The LOS link gain is given by [21]:

(1) $$H=\left\{\begin{array}{cc} \frac{\left(m+1\right)A_{r}}{2\pi d^{2}}\cos^{m}\left(\theta \right)\cos\left(\Psi \right)g\left(\Psi \right)T_{s}\left(\Psi \right) & ,0\leq \Psi \leq \Psi_{FOV}\\ 0 & ,\Psi >\Psi_{FOV} \end{array}\right.$$

where $A_{r}$ is the effective area of the Rx photodiode, *d* represents the distance between the Tx and the Rx, θ stands for the irradiance angle with respect to ***n_s_***, and Ψ is the incident angle with respect to ***n_r_*** (see Figure 1). $T_{s}\left(\Psi \right)$ is the optical filter gain, g(Ψ) the optical concentrator gain, $\Psi_{FOV}$ is the field of view (FOV) of the Rx and *m* represents the Lambertian emission, which is given by:

(2) $$m=\frac{-\ln(2)}{\ln(\cos\left(\theta_{1/2}\right))}$$

where $\theta_{1/2}$ is the half-power angle of the LED.

By adopting Lambert-Phong method [9], the diffuse paths are assumed to be represented by scattered rays, re-radiated from the wall to the Rx, which are being attenuated (i.e., based on the surface reflection coefficient). We define the reflection scattering using a generalized Lambert radiation pattern as:

(3) $$P_{rWall}=\frac{P_{i}\left(u+1\right)}{2\pi }\rho \cos^{u}\left(\delta \right)$$

where $P_{i}$ is the incident normalized unit power at the wall, $P_{rWall}$ is the reflection power from the reflected surface, *u* is the smoothness of the reflecting material, ρ is reflection coefficient, and δ is the randomly uniformly distributed angle between reflected rays and the diffusely reflected ray. Note that, in this paper, we study a practical scenario of VLC system with mobile users being used as RNs. To be as much as illustrative, we have used the average reflectivity over the entire visible spectrum defined by [22] and the nonlinearity of LED sources is not considered. However, model presented in this work can be extended to include non-LOS configuration (NLOS) (i.e., reflections) as part of the future studies, by considering spectral dependency of reflective surfaces [26] and non-Lambertian reflections [27,28].

### 3.2. Mobile User

**Figure 2:**
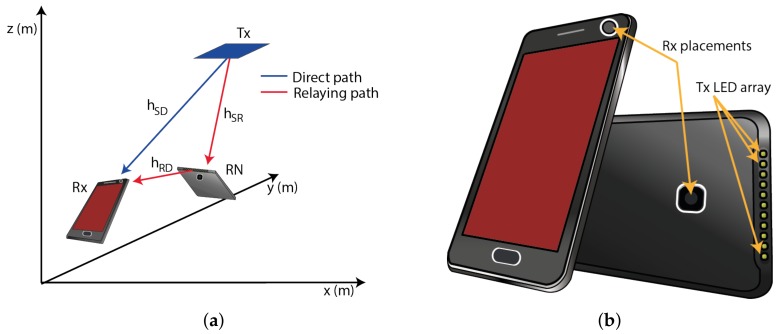
(**a**) Mobile user position in a room; and (**b**) the positions of the Rx and Tx on a mobile device.

Research work on direct VLC links using mobile devices as Rxs has been reported e.g., in [29]. In contrast to work reported in the literature, in this paper, we investigate the use of MP acting as the Rx and an RN as a part a relay-based VLC system, see Figure 2a. Let’s assume that the MP has (i) two photodetectors (PDs) on both sides, thus providing the MP with spatial diversity using a selection of the strongest received signal; and (ii) the Tx LED array placed perpendicular to the Rx planes as depicted in Figure 2b. Note that within the MP elevation plane, an azimuth angle remains the same as in the case of the Rx. The MP parameters are summarized in Table 2.

In this work we investigate the orientation of the MP within the indoor environment. Based on 1300 observations of people using their MPs on the street, airports, on trains and buses, 49% of them used their MPs with only one hand and up to 90% held it vertically facing upwards [30]. Based on our tests, people were reading messages and surfing the Internet by holding the MP typically with the elevation angle within the range of 5${}^{\circ }$–65${}^{\circ }$. Therefore, without any loss of generality, we have adopted the same elevations in this study. Note that, the download traffic (mostly data) is significantly higher than the upload and other forms of traffic as reported in [31], therefore we have focused only on the download case.

**Figure 3:**
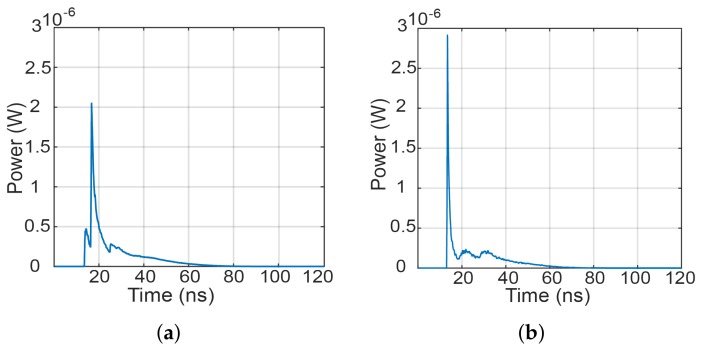
Impulse response of the link with MP acting as a Rx when using: (**a**) front camera; and (**b**) rear camera.

Let us have an example of a NLOS transmission when the Rx (i.e., the MP) is located near the corner of a room (i.e., the position of 0.5 m × 0.5 m × 1.2 m), see Figure 1b and Figure 2b. The upper edge of the user’s MP is oriented in azimuth and elevation angles of 180${}^{\circ }$ and 50${}^{\circ }$, respectively. The impulse responses of the link with no LOS path and using a MP-based Rx with front and rear cameras are depicted in Figure 3a,b, respectively. The impulse responses are calculated using the first five reflection components from walls. As can be seen from the figures, using the rear camera oriented to the Tx, the received power is higher and the impulse response is slightly less dispersive compared to the front photodiode.

### 3.3. Noise

At the Rx, there are three dominant noise sources: shot noise, thermal noise and intersymbol interference caused by an optical paths difference. The total noise variance is calculated as:

(4) $$\sigma_{total}^{2}=\sigma_{shot}^{2}+\sigma_{thermal}^{2}+\gamma^{2}P_{rISI}^{2}$$

where γ is the photodiode responsivity (A/W) and $P_{rISI}$ is the received power by intersymbol interference (ISI) given by:

(5) $$P_{rISI}=\int_{T}^{\infty }\left(h\left(t\right)⊗s\left(t\right)\right)dt$$

where h(t) is the impulse response, s(t) represents the transmitted optical pulse and the symbol ⊗ denotes convolution. The shot noise is defined in terms of its variance as [21]:

(6) $$\sigma_{shot}^{2}=2q\gamma \left(P_{r}+P_{rISI}\right)B+2qI_{bg}I_{2}B$$

where *q* is the electric charge, $P_{r}$ is the received optical power, *B* is the equivalent noise bandwidth, $I_{bg}$ is the background dark current and $I_{2}$ is the bandwidth noise factor. The thermal noise variance is independent of the incident power and is given by [21]:

(7) $$\sigma_{thermal}^{2}=\frac{8\pi kT_{k}}{G}\eta A_{r}I_{2}B^{2}+\frac{16\pi^{2}kT_{k}\Gamma }{g_{m}}\eta^{2}A_{r}^{2}I_{3}B^{3}$$

where the two terms represent feedback-resistor noise and field effect transistor (FET) channel noise, respectively. Here, *k* is the Boltzmann’s constant, $T_{K}$ is the absolute temperature, $I_{3}$ is the noise bandwidth factor, *G* is the open-loop voltage gain, η is the fixed capacitance of a PD per unit area, Γ is the FET channel noise factor and $g_{m}$ is the FET transconductance.

### 3.4. Modulation

Along with illumination, LEDs can be also used for data communications. Here, we have adopted the most common data format of on-off keying (OOK) for intensity modulation of LEDs. However, other modulation formats could also be used. The information bits of an LED are denoted by ${\left\{b^{j}\right\}}_{j=-\infty }^{\infty }$ where $b^{j}$ is a uniformly distributed sequence of {0,1}. The LED is ’on’ when $b^{j}=1$ and is ’off’ when $b^{j}=0$. Let rect(t) be a unit amplitude rectangular pulse of duration *T* (i.e., data rate $R_{d}=T^{-1}$). The transmitted optical signal is given by:

(8) $$s\left(t\right)=P_{p}\sum_{j=-\infty }^{\infty }b^{j}rect\left(t-jT\right)$$

where $P_{p}$ is the peak optical power of the emitted light wave. The received electrical signal at the photodiode is given by:

(9) y(t)=γh(t)⊗s(t-τ)+n(t)

where n(t) is the additive white Gaussian noise (AWGN) and τ denotes the transmission delay.

A standard matched filter is adopted at the Rx in order to recover the transmitted data. The impulse response of the filter at the Rx is a rectangular pulse of a unity amplitude and duration *T*. Let us assume τ=0, i.e., the matched filter of the Rx is synchronized to the arrival signal transmitted by an LED as in [24].

### 3.5. Relay Assisted Models

Among the various possible strategies available for user-based relay assisted cooperation [19,20], in this paper we have adopted: the AF and DF schemes. In this case, the source transmits a packet (or symbol) in one time slot and the RN re-transmits it in the next time slot, which are then combined at the destination prior to decision making. The scheme like in [18] consists of two phases. At first, the Tx sends data to both the relay and the Rx. In the relaying phase, the Tx remains silent and the relay terminal forwards the data to the Rx.

#### 3.5.1. Analytical Performance of AF Relaying

In the AF mode, the RN amplifies the received signal and forwards it to the Rx. Here we assume that the power of the signal retransmitted by the RN is scaled uniformly with respect to all bits in the packet with the average retransmission energy of $E_{S}$. In the 1st time slot/phase the sampled signals received at the RN ($y_{R}\left(t\right)$) and at the Rx (destination) ($y_{D}\left(t\right)$) are given by:

(10) $$y_{R}\left(t\right)=\sqrt{E_{s}}h_{SR}\left(t\right)⊗s\left(t\right)+n_{R}\left(t\right)$$

(11) $$y_{D}\left(t\right)=\sqrt{E_{s}}h_{SD}\left(t\right)⊗s\left(t\right)+n_{D}\left(t\right)$$

where $h_{SR}$ and $h_{SD}$ denote the VLC impulse responses for the Tx-RN and the Tx-Rx links, respectively, and $n_{R}$ and $n_{D}$ are AWGN noises. During the 2nd time slot/phase the signals at the output of the RN and received by the Rx are, respectively, given by [32]:

(12) $$x_{R}^{AF}\left(t\right)=\sqrt{\frac{E_{S}}{E_{S}h_{SR}^{2}\left(t\right)+\sigma_{total}^{2}}}h_{SR}\left(t\right)⊗s\left(t\right)+\sqrt{\frac{1}{E_{S}h_{SR}^{2}\left(t\right)+\sigma_{total}^{2}}}n_{R}\left(t\right)$$

(13) $$y_{D}^{AF}\left(t\right)=\sqrt{E_{s}}h_{RD}\left(t\right)⊗x_{R}^{AF}\left(t\right)+n_{D}^{{}^{\prime }}\left(t\right)$$

where $n_{D}^{{}^{\prime }}$ is the AWGN noise.

Combining (12) and (13), the *sampled signal* (from sampled signal we mean that the time varying signal is passed through a matched filter and it is sampled to maximize the signal-to-noise ratio, therefore, we drop the time index *t*) can be written as:

(14) $$y_{D}^{AF}=\sqrt{E_{s}}h_{RD}\sqrt{\frac{E_{S}h_{SR}^{2}}{E_{S}h_{SR}^{2}+\sigma_{total}^{2}}}s+\sqrt{E_{s}}h_{RD}\sqrt{\frac{1}{E_{S}h_{SR}^{2}+\sigma_{total}^{2}}}n_{R}+n_{D}^{{}^{\prime }}$$

From Equation (14) it is clear that $y_{D}^{AF}∼N\left(\mu_{1},\sigma_{1}^{2}\right)$ where $\mu_{1}=\sqrt{E_{s}}h_{RD}\sqrt{\frac{E_{S}h_{SR}^{2}}{E_{S}h_{SR}^{2}+\sigma_{total}^{2}}}s$ and $\sigma_{1}^{2}=\left(\frac{E_{s}h_{RD}^{2}}{E_{S}h_{SR}^{2}+\sigma_{total}^{2}}+1\right)\sigma_{total}^{2}$

**Log-Likelihood** **Detector**

At destination, the receiver has two copies of the transmitted signal. Employing the equal gain combining scheme at the Rx. The *sampled signal* is given as:

(15) $$y_{D}^{{}^{\prime }}=y_{D}+y_{D}^{AF}$$

It can be seen from (11) and (14) that the probability density function (PDF) of the sampled signal is given by:

(16) $$f\left(y_{D}^{{}^{\prime }}\right)=N\left(\mu_{2},\sigma_{2}^{2}\right)$$

where $\mu_{2}=\mu_{1}+\sqrt{E_{s}}h_{SD}s$ and $\sigma_{2}^{2}=\sigma_{1}^{2}+\sigma_{total}^{2}$.

The Rx will detect the transmitted bit from the received signal by using the log-likelihood ratio (LLR) detector, which can be written as:

(17) $$f\left(y_{D}^{{}^{\prime }}|s=1\right)\overset{1}{\underset{0}{≷}}f\left(y_{D}^{{}^{\prime }}|s=0\right)$$

Hence, for the AF cooperative scheme the LLR detector test gives:

(18) $$\frac{1}{\sqrt{2\pi \sigma_{2}^{2}}}e^{-\frac{{\left(y_{D}^{{}^{\prime }}-\mu_{2}^{{}^{\prime }}\right)}^{2}}{2\sigma_{2}^{2}}}\overset{1}{\underset{0}{≷}}\frac{1}{\sqrt{2\pi \sigma_{2}^{2}}}e^{-\frac{{\left(y_{D}^{{}^{\prime }}\right)}^{2}}{2\sigma_{2}^{2}}}$$

where $\mu_{2}^{{}^{\prime }}=\mu_{2}{|}_{s=1}$.

From (18), we get the following threshold-based detector, which indicates that if the value of received sampled signal is greater than the threshold $\kappa_{th}^{AF}$, then the transmitted symbol is estimated as 1, else it is 0:

(19) $$y_{D}^{{}^{\prime }}\overset{1}{\underset{0}{≷}}\kappa_{th}^{AF}$$

where

(20) $$\kappa_{th}^{AF}=\frac{1}{2}\left(\frac{E_{S}h_{SR}h_{RD}}{\sqrt{E_{S}h_{SR}^{2}+\sigma_{total}^{2}}}+\sqrt{E_{s}}h_{SD}\right)$$

**Bit Error** **Rate Calculation:**

The overall bit error probability of the considered VLC system with OOK is given as:

(21) $$P_{e}^{AF}=\frac{1}{2}\left(P_{e}\left(y_{D}^{{}^{\prime }}|s=0\right)+P_{e}\left(y_{D}^{{}^{\prime }}|s=1\right)\right)$$

Equation (21) can be rewritten as:

(22) $$P_{e}^{AF}=\frac{1}{2}\left(Pr\left(y_{D}^{{}^{\prime }}>\kappa_{th}^{AF}|s=0\right)+Pr\left(y_{D}^{{}^{\prime }}<\kappa_{th}^{AF}|s=1\right)\right)$$

where Pr(·) stands for the probability.

Employing (16) in (22), we get:

(23) $$P_{e}^{AF}=\frac{1}{2}\left(\int_{\kappa_{th}^{AF}}^{\infty }\frac{1}{\sqrt{2\pi \sigma_{2}^{2}}}e^{-\frac{{\left(y_{D}^{{}^{\prime }}\right)}^{2}}{2\sigma_{2}^{2}}}dy_{D}^{{}^{\prime }}+\int_{-\infty }^{\kappa_{th}^{AF}}\frac{1}{\sqrt{2\pi \sigma_{2}^{2}}}e^{-\frac{{\left(y_{D}^{{}^{\prime }}-\mu_{2}^{{}^{\prime }}\right)}^{2}}{2\sigma_{2}^{2}}}dy_{D}^{{}^{\prime }}\right)$$

Substituting $\frac{y_{D}^{{}^{\prime }}}{\sigma_{2}}=t$ and $\frac{y_{D}^{{}^{\prime }}-\mu_{2}^{{}^{\prime }}}{\sigma_{2}}=u$ in (21) we can rewrite it as:

(24) $$P_{e}^{AF}=\frac{1}{2}\left(\int_{\frac{\mu_{2}^{{}^{\prime }}}{2\sigma_{2}}}^{\infty }\frac{1}{\sqrt{2\pi }}e^{-\frac{t^{2}}{2}}dt+\int_{-\infty }^{-\frac{\mu_{2}^{{}^{\prime }}}{2\sigma_{2}}}\frac{1}{\sqrt{2\pi }}e^{-\frac{u^{2}}{2}}du\right)$$

Again substituting u=-v in the second integral of (24), we have:

(25) $$P_{e}^{AF}=\frac{1}{2}\left(\int_{\frac{\mu_{2}}{2\sigma_{2}}}^{\infty }\frac{1}{\sqrt{2\pi }}e^{-\frac{t^{2}}{2}}dt+\int_{\frac{\mu_{2}}{2\sigma_{2}}}^{\infty }\frac{1}{\sqrt{2\pi }}e^{-\frac{v^{2}}{2}}dv\right)$$

The integrals of (25) can be written in the form of Gaussian Q function as:

(26) $$P_{e}^{AF}=Q\left(\frac{\mu_{2}^{{}^{\prime }}}{2\sigma_{2}}\right)=Q\left(\frac{E_{S}h_{SR}h_{RD}+\sqrt{E_{s}}h_{SD}\sqrt{E_{S}h_{SR}^{2}+\sigma_{total}^{2}}}{2\sqrt{\left(E_{s}h_{RD}^{2}+2\left(E_{S}h_{SR}^{2}+\sigma_{total}^{2}\right)\right)\sigma_{total}^{2}}}\right)$$

#### 3.5.2. Analytical Performance of Selective DF Relaying

In the DF scheme the source will transmit the signal to the both relay and the destination within the 1st time slot. Here, the relay will follow the selective DF cooperative scheme. If the relay decodes the signal correctly then it will retransmit the signal to the destination during the 2nd time slot/phase, otherwise it will stay idle. The received signal in the DF scheme is given by:

(27) $$y_{D}^{DF}\left(t\right)=\sqrt{E_{s}}h_{RD}\left(t\right)⊗s\left(t\right)+n_{D}^{{}^{\prime }}\left(t\right)$$

The equations for the signal transmitted by the source to the relay and from the source to the destination are same as (10) and (11).

Due to selective relaying, the received sampled signal at the destination is given by:

(28) $$y_{D}^{{}^{\prime }}=\left\{\begin{array}{c} y_{D}^{DF}+y_{D},\;\;\mathrm{when}\;\mathrm{relay}\;\mathrm{decodes}\;\mathrm{correctly}\\ y_{D},\;\;\mathrm{when}\;\mathrm{relay}\;\mathrm{does}\;\mathrm{not}\;\mathrm{decode}\;\mathrm{correctly} \end{array}\right.$$

It can be easily verified from (28) that when relay decodes correctly, then we have:

(29) $$y_{D}^{{}^{\prime }}∼N\left(0,2\sigma_{total}^{2}\right),\;\;for\;\;s=0$$

(30) $$y_{D}^{{}^{\prime }}∼N\left(\left(h_{SD}+h_{RD}\right)\sqrt{E_{s}},2\sigma_{total}^{2}\right),\;\;for\;\;s=1$$

From (29) and (30) the LLR detection rule can be written as:

(31) $$\frac{1}{\sqrt{4\pi \sigma^{2}}}e^{-\frac{{\left(y_{D}^{{}^{\prime }}\right)}^{2}}{4\sigma^{2}}}\overset{0}{\underset{1}{≷}}\frac{1}{\sqrt{4\pi \sigma^{2}}}e^{-\frac{{\left(y_{D}^{{}^{\prime }}-\sqrt{E_{s}}\left(h_{SD}+h_{RD}\right)\right)}^{2}}{4\sigma^{2}}}$$

Solving (31) results in the following detection condition:

(32) $$y_{D}^{{}^{\prime }}\overset{1}{\underset{0}{≷}}\frac{\sqrt{E_{s}}\left(h_{SD}+h_{RD}\right)}{2}=\kappa_{th}^{DF,1}$$

Similarly, when the relay is in error and remains idle, we have the following detection condition:

(33) $$y_{D}^{{}^{\prime }}\overset{1}{\underset{0}{≷}}\frac{\sqrt{E_{s}}h_{SD}}{2}=\kappa_{th}^{DF,0}$$

Based on (32) and (33), the destination uses the following detection [33]:

(34) $$y_{D}+vy_{D}^{DF}\overset{1}{\underset{0}{≷}}\kappa_{th}^{DF,v}$$

where v=1 when relay transmits and v=0 when relay does not transmit.

The BER for the considered VLC system for the case *when the relay is transmitting* can be given as:

(35) $$P_{e}^{DF,1}=\frac{1}{2}\left(\int_{\kappa_{th}^{DF,1}}^{\infty }\frac{1}{\sqrt{4\pi \sigma_{total}^{2}}}e^{-\frac{{\left(y_{D}^{{}^{\prime }}\right)}^{2}}{4\sigma_{total}^{2}}}dy_{D}^{{}^{\prime }}+\int_{-\infty }^{\kappa_{th}^{DF,1}}\frac{1}{\sqrt{4\pi \sigma_{total}^{2}}}e^{-\frac{{\left(y_{D}^{{}^{\prime }}-\sqrt{E_{s}}\left(h_{SD}+h_{RD}\right)\right)}^{2}}{4\sigma_{total}^{2}}}dy_{D}^{{}^{\prime }}\right)$$

Solving (35) in a similar way as (23) the BER is given as:

(36) $$P_{e}^{DF,1}=Pr\left(y_{D}+y_{D}^{DF}<\kappa_{th}^{DF,1}|s=1\right)=Q\left(\frac{\sqrt{E_{s}}\left(h_{SD}+h_{RD}\right)}{2\sqrt{2}\sigma_{total}}\right)$$

Similarly, the BER for the case when the relay is in error and remains idle in the 2nd phase can be found as:

(37) $$P_{e}^{DF,0}=Pr\left(y_{D}<\kappa_{th}^{DF,0}|s=1\right)=Q\left(\frac{\sqrt{E_{s}}h_{SD}}{2\sqrt{2}\sigma_{total}}\right)$$

Further, the BER of the relay is given by:

(38) $$P_{e}^{R}=Q\left(\frac{\sqrt{E_{s}}h_{SR}}{2\sqrt{2}\sigma_{total}}\right)$$

Using (36)–(38), and results given in [33], the overall BER for the proposed VLC system using the selective DF cooperative scheme is given as:

(39) $$P_{e}^{DF}=Q\left(\frac{\sqrt{E_{s}}h_{SR}}{2\sqrt{2}\sigma_{total}}\right)Q\left(\frac{\sqrt{E_{s}}h_{SD}}{2\sqrt{2}\sigma_{total}}\right)+\left(1-Q\left(\frac{\sqrt{E_{s}}h_{SR}}{2\sqrt{2}\sigma_{total}}\right)\right)Q\left(\frac{\sqrt{E_{s}}\left(h_{SR}+h_{RD}\right)}{2\sqrt{2}\sigma_{total}}\right)$$

**Figure 4:**
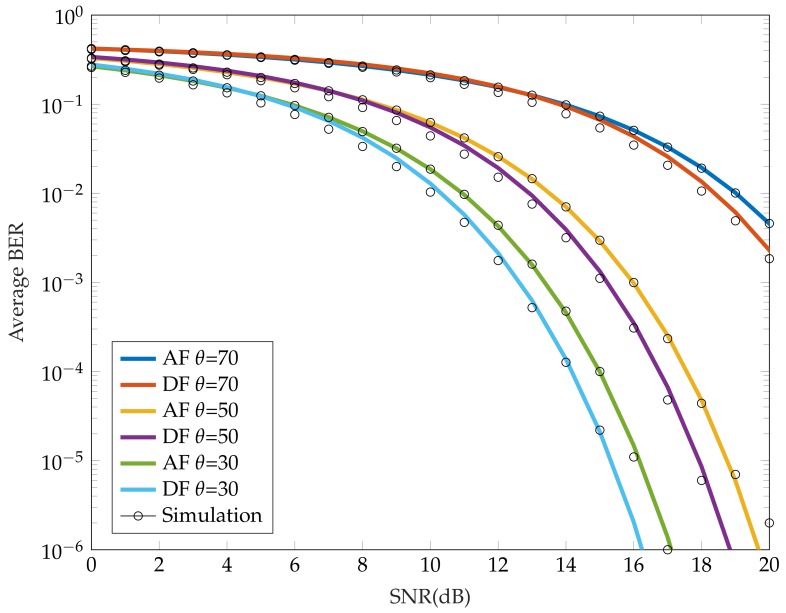
Comparison of average BER for different irradiance angle with constant FOV.

Figure 4 provides a comparison of the average BER as a function of SNR for both AF and DF schemes and for different irradiance angles of the relay and the source. The mobile parameters were used from Table 2, a distance between the source and the end user was set to 3 m and the RN was located in the middle of the link. We can clearly see how DF outperforms AF. As the value of irradiance angle increases for a constant FOV, the performance of the considered VLC system degrades. For example at a $BER=10^{-3}$ the power penalties are 0.8 dB and 0.75 dB for θ = 30${}^{\circ }$ and 50${}^{\circ }$, respectively.

## 4. Simulation Results

In this section, we present the results for the performance analyses of the proposed VLC relay cooperation system. In order to provide a more accurate comparison between AF and DF modes, we have adopted a simulation model with 5 reflections based on the Monte Carlo ray tracing algorithm using the assumption of a half-duplex OOK cooperation transmission link. For simulations, we have used the key parameters shown in Table 2.

**Figure 5:**
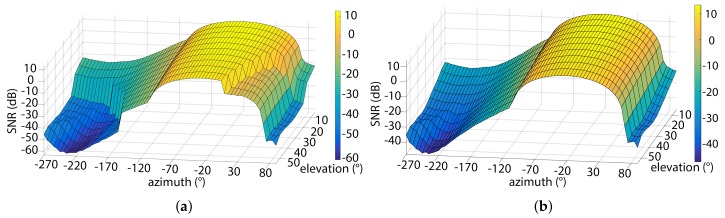
SNR dependency on azimuthal orientation of relay in: (**a**) AF; and (**b**) DF.

In order to evaluate the azimuthal and angular dependency of the RN, we assessed a scenario where the relay user is located at the coordinates of 2 m × 2 m × 1.2 m with the transmit power of 2 W. Figure 5a,b depict a comparison of the SNR as a function of the azimuth and elevation angles for AF and DF relaying schemes. SNR > 9.8 dB corresponds to a BER of 10^-3^ for the relay user transmission. Note that, the maximum SNR is achieved at the azimuth angle of –15${}^{\circ }$.

**Figure 6:**
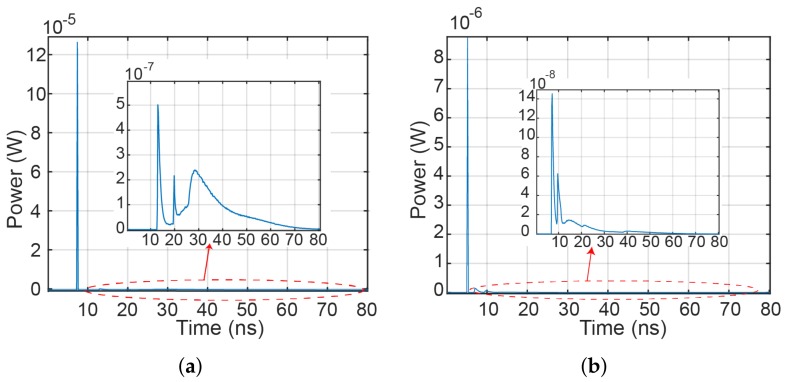
Impulse response for VLC with RN for: (**a**) source to RN; and (**b**) RN to the Rx.

To illustrate the position of the mobile relay user within the room, see Figure 1b. We calculated the impulse response of the channel, considering that the mobile relay user can only move around within a specific region in the room, see yellow marked area in Figure 1b. As an example for the relay-assisted DF model, with the RN positioned at the coordinates of 2 m × 2 m × 1.2 m with azimuth and elevation angles of –20${}^{\circ }$ and 5${}^{\circ }$, respectively, the impulse responses for source-to-RN and RN-to-Rx are depicted in Figure 6a,b, respectively. The channel gains for the source-relay $G_{SR}$ and the relay-user $G_{RD}$ links against the direct source-user link are determined to be 39.1 dB and 4.4 dB, respectively.

**Figure 7:**
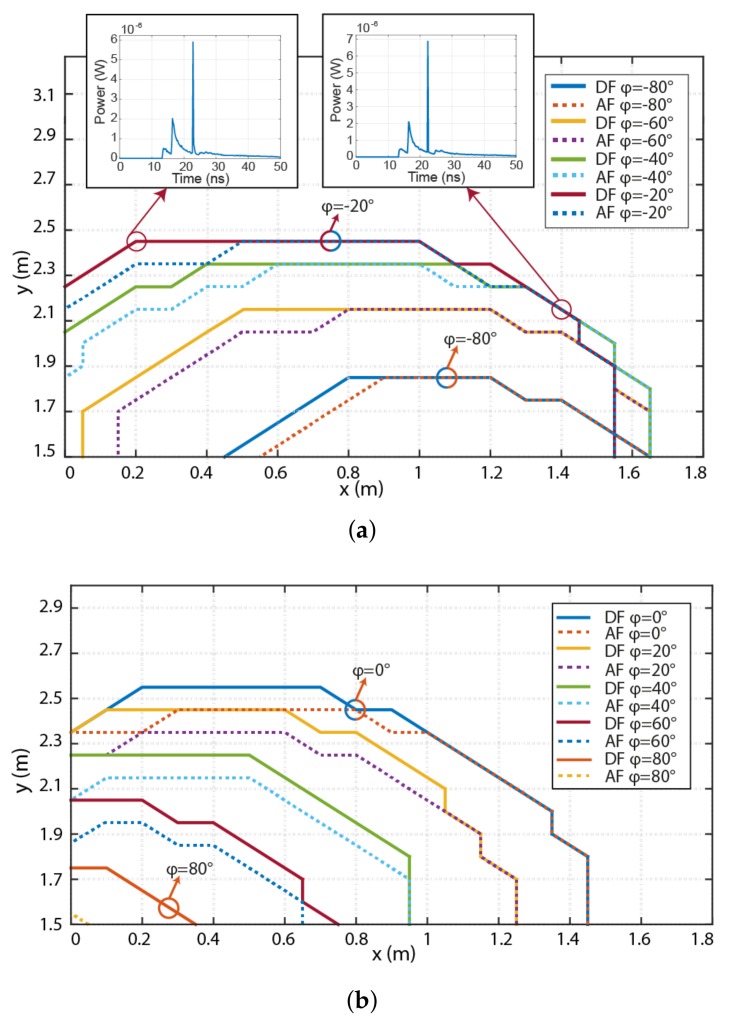
Azimuthal dependency of the RN with an elevation angle of 5${}^{\circ }$ with RN oriented toward the: (**a**) right wall; and (**b**) left wall. Curves show the borders where the RN can be used and ensures a BER of < 10^–3^ for the entire link. Insets in (**a**) illustrate impulse responses of the complete relay-assisted link.

Next we considered the azimuthal orientation of the RN against its position within the yellow area of the room, see Figure 1b. In the simulation, we have assumed that the Rx is (i) at the elevation angle of 55${}^{\circ }$; and (ii) an azimuth angle is –180${}^{\circ }$. Figure 7 shows the borders of the room covered by the RN for a range of azimuthal angles to ensure a BER of 10^–3^. Figure 7a is for the case when the RN is oriented more to the opposite direction from the source (negative azimuth). It can be seen that the DF mode offers improved results more specifically at positions further from the source (i.e., *x* = 0 to 0.8) and close to the wall. For the azimuth of –20${}^{\circ }$, with the DF the coverage area is only increased by ∼0.2 m${}^{2}$ compared to AF, therefore less complex AF would be the preferred option to adopt. A difference of 20${}^{\circ }$ in the azimuthal plane results in changes in the coverage area by ∼30 cm and 40 cm in the *x*- and *y*-axis, respectively. Note that, RN widens the coverage area by ∼1.9 m${}^{2}$ for the azimuth angle changed from –80${}^{\circ }$ (orientation to the wall) to –20${}^{\circ }$. The insets in Figure 7a depict the overall impulse responses of the VLC channel (i.e., from Tx to the Rx via the RN) for given positions and the azimuth of –20${}^{\circ }$, where SNR is mainly affected by the ISI.

The azimuthal orientation of the RN in the contra-clockwise direction (i.e., from the 0${}^{\circ }$ to 80${}^{\circ }$ towards the Tx) is illustrated in Figure 7b. In the case where the RN rotates in azimuth to the left, the DF cooperative mode offers an improvement of more than 10 cm for all positions in the *y*-axis. Note that, the relay MP azimuthal oriented in 80${}^{\circ }$ can be used only in small fraction area. For a wider angle of rotation, the difference in the coverage area between AF and DF modes increases from 0.2 m^2^ for 20${}^{\circ }$ to 0.4 m^2^ for 60${}^{\circ }$. Whereas, the azimuthal orientations of 0${}^{\circ }$ and 80${}^{\circ }$ result in widening of the coverage area by 2.16 m^2^.

**Figure 8:**
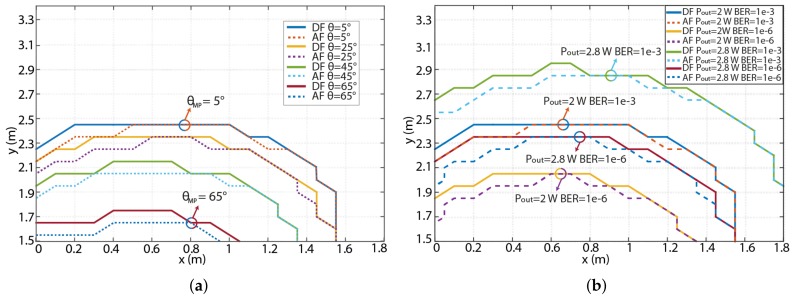
(**a**) Elevation angle dependence of RN for both AF and DF-based links for a range of irradiance angle $\theta_{MP}$, an azimuth angle of –20${}^{\circ }$ and a BER of < 10^–3^; and (**b**) RN transmitted power and the BER profiles for both DF and AF-based links.

In the following, we show how the RN area changes based on the elevation of the MP for both AF and DF-based links for a range of elevation angles $\theta_{MP}$, an azimuth angle of –20${}^{\circ }$, and a BER of < 10^–3^ as illustrated in Figure 8a. The maximum covered area is achieved for $\theta_{MP}$ of 5${}^{\circ }$, therefore RN can be placed up to 2 m from the Rx. Increasing $\theta_{MP}$ to 25${}^{\circ }$ results in the reduced distance between the RN and Rx by ∼10 cm. For $\theta_{MP}$ of 65${}^{\circ }$ the maximal RN position in the *y*-axis is only 1.6 m. Note that, for 25${}^{\circ }$<$\theta_{MP}$< to 45${}^{\circ }$ the coverage area is changed by 0.67 m^2^.

The final result illustrates how the RN area can be extended either by increasing the transmit power $P_{out}$ (i.e., more LEDs on the MP) at the RN or by changing the target BER. In Figure 8b, we compare the transmit power from the relay MP for both DF and AF modes for the optimum azimuth and elevation angles of –20${}^{\circ }$ and 5${}^{\circ }$, respectively. For example, for a BER of 10${}^{-3}$ using a LED array of 1 × 14 with the $P_{out}$ of ∼2.8 W the coverage area for relay-assisted communications is increased by ∼1.43 m${}^{2}$ compared to the LED array of 1 × 10. Note that, in case of $P_{out}$ of 2 W and lower BER target 10${}^{-6}$ we can observe reduced coverage area as expected.

## 5. Conclusions

In this paper, we investigated an OOK half-duplex-based VLC link with a mobile unit-based relay node used to improve the link availability and coverage area in a typical office environment. For the first time, the real mobile was considered with two photodetectors on both sides of mobile phone (utilising spatial diversity) and a perpendicular placed transmitting LED array. We considered the case where the receiver was positioned close to the corner of the room and we investigated the optimal position of the relay node based on its azimuthal and elevation orientation. The results showed significant improvement in the link performance using cooperative schemes when compared to direct NLOS transmission. In addition, we derived analytic model that compared DF and AF relay techniques. The results showed that DF outperforms the AF relaying scheme for different irradiance angles. The power penalties at a BER of 10${}^{-3}$ were 0.8 and 0.75 dB for θ = 30${}^{\circ }$ and 50${}^{\circ }$. Numerical results also illustrated that the DF relay-based system offered a wider coverage area compared with the AF scheme.

## Figures and Tables

**Table 1:** Key System Parameters.

Parameter	Symbol	Value
Room size	-	5 × 5 × 3 m
No. of rays	-	100,000
No. of reflection	-	5
Time resolution	Δt	0.2 ns
Bit rate	-	100 Mb/s
Reflectivity of walls	$\rho_{wall}$	0.74
Reflectivity of ceiling	$\rho_{ceiling}$	0.38
Reflectivity of floor	$\rho_{floor}$	0.61
Smoothness of the reflecting material	*u*	1
Tx position	-	2.5 × 2.5 × 2.8 m
Tx power per LED	-	20 mW
Size of the LED array	-	60 × 60
Semiangle at half power	$\theta_{1/2}$	60${}^{\circ }$
Tx elevation	-	–90${}^{\circ }$
Tx azimuth	-	0${}^{\circ }$

**Table 2:** Mobile Device Parameters.

Parameter	Symbol	Value
Rx area	$A_{r}$	1 cm${}^{2}$
Effective area of a photodiode	$\Psi_{FOV}$	50${}^{\circ }$
Photodetector responsivity	γ	0.53 A/W
Optical filter concentrator	Ts	1
Optical concentrator gain	*g*	3
User position	-	0.5 × 0.5 × 1.2 m
Rx elevation	-	50${}^{\circ }$
Rx azimuth	-	90${}^{\circ }$
Tx_RN_ power per LED	-	200 mW
Size of LEDs	-	1 × 10, 1 × 14
Semiangle at half power	$\theta_{1/2}$	60${}^{\circ }$
Background dark current	$I_{bg}$	10 nA
Noise bandwidth factors	$I_{2}$, $I_{3}$	0.562, 0.0868
Absolute temperature	$T_{k}$	295 K
Open-loop voltage gain	*G*	10
Capacitance	η	112 × 10^-8^ F/m^2^
FET channel noise factor	Γ	1.5
FET transconductance	$g_{m}$	0.03 S
